# Association of surfactant protein A polymorphisms with otitis media in infants at risk for asthma

**DOI:** 10.1186/1471-2350-7-68

**Published:** 2006-08-02

**Authors:** Melinda M Pettigrew, Janneane F Gent, Yong Zhu, Elizabeth W Triche, Kathleen D Belanger, Theodore R Holford, Michael B Bracken, Brian P Leaderer

**Affiliations:** 1 Yale Center for Perinatal, Pediatric and Environmental Epidemiology, Department of Epidemiology and Public Health, Yale University School of Medicine, New Haven, CT, USA

## Abstract

**Background:**

Otitis media is one of the most common infections of early childhood. Surfactant protein A functions as part of the innate immune response, which plays an important role in preventing infections early in life. This prospective study utilized a candidate gene approach to evaluate the association between polymorphisms in loci encoding SP-A and risk of otitis media during the first year of life among a cohort of infants at risk for developing asthma.

**Methods:**

Between September 1996 and December 1998, women were invited to participate if they had at least one other child with physician-diagnosed asthma. Each mother was given a standardized questionnaire within 4 months of her infant's birth. Infant respiratory symptoms were collected during quarterly telephone interviews at 6, 9 and 12 months of age. Genotyping was done on 355 infants for whom whole blood and complete otitis media data were available.

**Results:**

Polymorphisms at codons 19, 62, and 133 in SP-A1, and 223 in SP-A2 were associated with race/ethnicity. In logistic regression models incorporating estimates of uncertainty in haplotype assignment, the 6A^4^/1A^5^haplotype was protective for otitis media among white infants in our study population (OR 0.23; 95% CI 0.07,0.73).

**Conclusion:**

These results indicate that polymorphisms within SP-A loci may be associated with otitis media in white infants. Larger confirmatory studies in all ethnic groups are warranted.

## Background

Otitis media is one of the most common infections of early childhood: 60%-80% of children will have at least one episode during their first year of life [1,2]. Rates of early-onset (before 6 months of age) and recurrent otitis media are increasing in the United States [3,4]. Recurrent otitis media may increase a child's risk for hearing loss and delays in speech development [1,5].

Acute otitis media is an infectious disease resulting from complex interactions between microbes, the environment, and the host immune response. The majority of episodes are thought to be bacterial, but acute otitis media is most often associated with, or preceded by, a viral respiratory infection [6,7]. Epidemiologic studies have consistently highlighted the importance of daycare attendance and the number of siblings as predictors of acute otitis media [6,7]. Genetic factors play an important role in otitis media susceptibility. Monozygotic twins have higher concordance in otitis media rates than dizygotic twins, and estimates of heritability range from 0.45–0.74 [8-10]. While studies have provided strong evidence of a genetic predisposition to otitis media, only a few studies have identified specific genes related to otitis media risk [11,12].

The innate immune response may be critical for otitis media susceptibility in early life before the acquisition of specific immunity. Surfactant protein A (SP-A) is the most abundant surfactant protein in the lung, and is an important component of the innate immune system [13]. SP-A is a member of the collectin protein family; these proteins recognize carbohydrates on the surface of pathogens via the carbohydrate recognition domain [14]. As a pattern recognition receptor, SP-A functions in the first line of defense in the absence of specific antimicrobial antibodies. SP-A knockout mice show delayed clearance of otitis media associated pathogens *Haemophilus influenzae *and respiratory syncitial virus (RSV) [15]. The gene locus for SP-A is in chromosome 10q21 through q24 and has two functional and highly homologous SP-A genes (SP-A1 and SP-A2) [16-18].

SP-A has been detected in the Eustachian tube [19] and may play a role in the pathogenesis of otitis media [20]. Recurrent otitis media has previously been linked to the 6A^4^/1A^5 ^haplotype in SP-A [11]. This study took place in Finland, and the distribution of SP-A haplotypes in the Finnish population has been shown to differ from frequencies found within the United States [21]. Among a prospective cohort of infants at risk for developing asthma, we used a candidate gene approach to determine whether polymorphisms within the SP-A1 and SP-A2 genes were associated with any otitis media during the first year of life.

## Methods

### Cohort

Data used in this report were obtained from infants enrolled in a longitudinal cohort to study the impact of environmental exposures on asthma development and morbidity. Between September 1996 and December 1998, women delivering babies in six hospitals were invited to participate if they had at least one other child at home younger than 12 years of age with physician-diagnosed asthma. Because of the asthmatic sibling, these infants were considered at risk for this disease. A full description of the methods is provided elsewhere [22-24]. Our Human Investigations Committee as well as institutional review boards at each participating hospital reviewed and approved the study.

Of the initial 1,002 infants enrolled, data were available on otitis media during the first year of life for 889. Between the third and fifth year of participation, a second visit was made to the home at which time an effort was made to collect a blood sample from the cohort subjects. The analyses in this report include a convenience sample of 355 infants for whom whole blood and complete quarterly otitis media data were available.

### Data collection

Trained research assistants described the study to the infant's mother, obtained written informed consent, and administered standardized questionnaires to study participants during home interviews within 4 months of the infant's birth. We collected household demographic data such as maternal race and ethnicity, education, and number of children. We also collected detailed information regarding infant care such as use of daycare, any breastfeeding during the first year of life, and whether the infant was exposed to environmental tobacco smoke (ETS) in the home. Mothers were asked to report on their history of allergies or physician-diagnosed asthma. Infant respiratory symptoms were collected during quarterly telephone interviews at 6, 9 and 12 months of age. During these telephone interviews, we asked the mother to report her infant's respiratory symptoms and doctor or clinic visits (month and year of visit, reason for visit, and mother's report of the diagnosis). When the infant reached one year of age, each mother completed an extensive phone questionnaire covering her infant's health status during the previous year.

### Definitions of otitis media

Otitis media was ascertained during each quarterly survey by mother's report of an episode of clinician-diagnosed otitis media with the month and year of diagnosis. Additional physician diagnoses of otitis media occurring in the same month or next consecutive month were counted as a single episode.

### Genotyping of SP-A genes

DNA from whole blood samples was extracted using the QIAamp DNA blood minikit (Qiagen) according to the manufacturer's instructions. Genotyping for the single nucleotide polymorphisms (SNPs) at aa19, aa50, aa62, aa133, and aa219 in SP-A1 and aa9, aa91, aa223 in SP-A2 were done using a sequence specific primer-PCR methodology described by Pantelidis et al.[25]. This method involves high stringency PCR amplification using allele-specific primers designed with the final 3' end nucleotide complementary to the nucleotide variants within a particular SNP locus. In our hands, this method did not give reproducible results for the CT polymorphism at aa140 within SP-A2. To detect polymorphisms in SP-A2 at aa140, we used PCR based cRFLP as described by DiAngelo et al. [26].

### Data analysis

Unadjusted associations between otitis media episodes and selected study characteristics, SNPs, and individual SP-A1 or -A2 alleles were initially evaluated by χ^2 ^tests. Observed SNP frequencies were tested for Hardy-Weinberg equilibrium by χ^2 ^analysis. Allele haplotypes for SP-A1 and for SP-A2 were examined for linkage disequilibrium and haplotype frequencies were estimated using PROC HAPLOTYPE in the SAS/Genetics module of SAS version 9.1 (Cary, NC). Logistic regression was used to model the association between SP-A1 and SP-A2 haplotypes and otitis media. Note that specific haplotypes can only be inferred since each individual has two chromosomes, and the methods of genotyping provide only the two bases at each site without a chromosomal assignment. For example, homozygous SNPs result in one and only one haplotype assignment. In the presence of heterozygous SNPs, the allele haplotype could be one of several, each occurring with a probability based on observed SNP frequencies. Therefore, we made an effort to estimate the effect of uncertainty in haplotype assignment on model variability. We used the set of potential haplotypes (PROC HAPLOTYPE) and a regression calibration technique similar to that described by Carroll et al. [27] to create 100 separate data sets by randomly assigning a haplotype for a particular subject based on the probability of each of the possible haplotypes given for that subject. Next, 100 separate logistic regression models with coefficient and variance estimates for the i^th ^model of b_i _and σi2
 MathType@MTEF@5@5@+=feaafiart1ev1aaatCvAUfKttLearuWrP9MDH5MBPbIqV92AaeXatLxBI9gBaebbnrfifHhDYfgasaacH8akY=wiFfYdH8Gipec8Eeeu0xXdbba9frFj0=OqFfea0dXdd9vqai=hGuQ8kuc9pgc9s8qqaq=dirpe0xb9q8qiLsFr0=vr0=vr0dc8meaabaqaciaacaGaaeqabaqabeGadaaakeaaiiGacqWFdpWCdaqhaaWcbaGaemyAaKgabaGaeGOmaidaaaaa@30F0@, respectively, were fit for each haplotype of interest. The estimate of the association (β^
 MathType@MTEF@5@5@+=feaafiart1ev1aaatCvAUfKttLearuWrP9MDH5MBPbIqV92AaeXatLxBI9gBaebbnrfifHhDYfgasaacH8akY=wiFfYdH8Gipec8Eeeu0xXdbba9frFj0=OqFfea0dXdd9vqai=hGuQ8kuc9pgc9s8qqaq=dirpe0xb9q8qiLsFr0=vr0=vr0dc8meaabaqaciaacaGaaeqabaqabeGadaaakeaaiiGacuWFYoGygaqcaaaa@2E64@) between otitis media and each particular haplotype was calculated as the mean of the 100 logistic regressions. The variability of the estimate was calculated as

var (β^
 MathType@MTEF@5@5@+=feaafiart1ev1aaatCvAUfKttLearuWrP9MDH5MBPbIqV92AaeXatLxBI9gBaebbnrfifHhDYfgasaacH8akY=wiFfYdH8Gipec8Eeeu0xXdbba9frFj0=OqFfea0dXdd9vqai=hGuQ8kuc9pgc9s8qqaq=dirpe0xb9q8qiLsFr0=vr0=vr0dc8meaabaqaciaacaGaaeqabaqabeGadaaakeaaiiGacuWFYoGygaqcaaaa@2E64@) = mean (σi2
 MathType@MTEF@5@5@+=feaafiart1ev1aaatCvAUfKttLearuWrP9MDH5MBPbIqV92AaeXatLxBI9gBaebbnrfifHhDYfgasaacH8akY=wiFfYdH8Gipec8Eeeu0xXdbba9frFj0=OqFfea0dXdd9vqai=hGuQ8kuc9pgc9s8qqaq=dirpe0xb9q8qiLsFr0=vr0=vr0dc8meaabaqaciaacaGaaeqabaqabeGadaaakeaaiiGacqWFdpWCdaqhaaWcbaGaemyAaKgabaGaeGOmaidaaaaa@30F0@) + var (b_i_s)

where mean (σi2
 MathType@MTEF@5@5@+=feaafiart1ev1aaatCvAUfKttLearuWrP9MDH5MBPbIqV92AaeXatLxBI9gBaebbnrfifHhDYfgasaacH8akY=wiFfYdH8Gipec8Eeeu0xXdbba9frFj0=OqFfea0dXdd9vqai=hGuQ8kuc9pgc9s8qqaq=dirpe0xb9q8qiLsFr0=vr0=vr0dc8meaabaqaciaacaGaaeqabaqabeGadaaakeaaiiGacqWFdpWCdaqhaaWcbaGaemyAaKgabaGaeGOmaidaaaaa@30F0@) is defined as the mean of the 100 logistic regression variances and var (b_i_s) is defined as the variance of the 100 logistic regression b coefficients.

## Results

### Participant characteristics

The majority of the 355 infants in our study experienced otitis media during their first year. Thirty-eight percent of the infants experienced only one episode of otitis media, 24% experienced two, and 10% had three or more episodes during their first year of life (Table 1). Male infants were more likely to experience otitis media during their first year of life: 78% of males experienced otitis media compared to 67% of female infants (P = 0.02). Black infants tended to have less otitis media than white or Hispanic infants during their first year of life. For example, 58% of black infants experienced otitis media compared to 77% of white infants and 73% of Hispanic infants (P = 0.06). We did not find significant associations between maternal education, maternal allergies, maternal asthma, daycare before six months of age, breastfeeding, exposure to ETS in the home, or season of birth and otitis media during the first year of life.

**Table 1:** Unadjusted associations between personal characteristics and otitis media episodes^a ^for 355 infants at risk for developing asthma. (CT and MA, 1998 – 2000)

		**Otitis Media Episodes**			
		
		**0**	**1**	**2**	**3+**
		
**Characteristic**	**n (%)**	**%**	**%**	**%**	**%**
**All subjects N (%)**	**355**	**97 (27.3)**	**137 (38.6)**	**87 (24.5)**	**34 (9.6)**
**Gender**					
Male	171 (48.2)	21.6	42.7	24.6	11.1
Female	184 (51.8)	32.6	34.8	24.5	8.2
**Ethnicity**					
White	226 (63.7)	23.4	38.0	28.8	9.7
Black	48 (13.5)	41.7	37.5	14.6	6.2
Hispanic	62 (17.5)	27.4	41.9	22.6	8.1
Other	19 (5.4)	36.8	36.8	5.3	21.0
**Maternal education**					
< HS	38 (10.7)	29.0	36.8	29.0	5.3
Some college	179 (50.4)	29.0	39.7	20.1	11.2
College degree	138 (38.9)	24.6	37.7	29.0	8.7
**Maternal allergies**					
No	146 (41.1)	30.8	39.7	24.0	5.5
Yes	209 (58.9)	24.9	37.8	24.9	12.4
**Maternal asthma**					
No	246 (69.3)	30.1	38.2	22.0	9.8
Yes	109 (30.7)	21.1	39.4	30.3	9.2
**Daycare before 6 mo**					
No	279 (78.6)	29.0	39.4	23.3	8.2
Yes	76 (21.4)	21.0	35.5	29.0	14.5
**Breast feeding**					
No	113 (31.8)	27.4	40.7	22.1	9.7
Yes	242 (68.2)	27.3	37.6	25.6	9.5
**ETS**					
No	304 (86.6)	26.0	38.5	26.3	9.2
Yes	47 (13.4)	34.0	40.4	14.9	10.6
**Season of birth**					
Winter	75 (21.1)	28.0	37.3	26.7	8.0
Spring	101 (28.4)	25.7	41.6	24.8	7.9
Summer	97 (27.3)	26.8	39.2	27.8	6.2
Fall	82 (23.1)	29.3	35.4	18.3	17.1

^a^Otitis media determined by mother's report. More than one episode reported in a month or in the next consecutive month counted as a single episode.

There were socioeconomic differences between the group from the study cohort who chose to participate in the blood draw (355 out of 889 [40%]) and those who did not (n = 554): whole blood samples were obtained from more white infants (64% vs. 57%) and fewer Hispanic infants (18% vs. 26%) (P = 0.04), more of the mothers in the blood draw group had college degrees (39% vs. 31%, P = 0.03) and chose to breastfeed their infants (68% vs. 60%, P = 0.02). The rates of otitis media between the two blood draw groups did not differ: 28% compared to 26% for otitis media in the first 6 months; 73% vs. 71% for any otitis media in the first year; and 10% vs. 9% for 3 or more episodes of otitis media in the first year for the blood draw participants vs. non-participants, respectively.

### Analysis of single nucleotide polymorphisms

Table 2 contains unadjusted associations between each of the nine candidate SNPs and otitis media for all 355 infants in our sample. Each of the individual SNPs was in Hardy-Weinberg equilibrium in the full sample of 335 subjects and within each racial/ethnic category (P > 0.05). Polymorphisms in SP-A1 at codon 19 were associated with otitis media during the first year of life. Infants with an alanine (CT) at codon 19 were also more likely to have a greater number of otitis media episodes during their first year of life. The percentage of infants with an alanine at codon 19 and 0, 1, and 2 or more episodes of otitis media during their first year were 10%, 49%, and 41% respectively. In contrast, the percentage of infants with valine (homozygote TT) at codon 19 and 0, 1, and 2 or more episodes of otitis media during their first year were 31%, 36%, and 33% respectively (P = 0.01).

**Table 2:** Unadjusted associations between SNPs from surfactant protein A alleles (SP-A1, SP-A2) and otitis media (OM) episodes before 12 months of age for 355 infants at risk for developing asthma. (CT and MA, 1998 – 2000)

		**OM Episodes**
		
**SNP**	**n (%)**	**%**
**All subjects N (%)**	**355**	**258 (72.7)**
**SP-A1^a^**		
**aa 19^b^**		
CC	1 (0.3)	
CT	56 (15.8)	89.5
TT	294 (83.8)	69.0
**aa 50**		
CC	75 (21.2)	80.0
CG	171 (48.3)	69.0
GG	108 (30.5)	74.1
**aa 62**		
GG	5 (1.4)	
AG	88 (25.2)	74.2
AA	256 (73.4)	71.9
**aa 133**		
GG	2 (0.6)	
AG	51 (14.4)	83.0
AA	302 (85.1)	70.9
**aa 219**		
TT	1 (0.3)	
CT	46 (13.0)	68.1
CC	308 (86.8)	73.4
**SP-A2^a^**		
**aa 9**		
CC	73 (20.6)	74.0
AC	171 (48.2)	71.9
AA	111 (31.3)	73.0
**aa 91**		
CC	6 (1.7)	
CG	85 (24.2)	73.6
GG	261 (74.2)	72.4
**aa 140**		
TT	25 (7.1)	64.0
CT	142 (40.2)	71.1
CC	186 (52.7)	75.3
**aa 223**		
AA	18 (5.1)	77.8
AC	129 (36.3)	72.9
CC	208 (58.6)	72.1

^a^Each of the SNPs was in Hardy-Weinberg equilibrium (P > 0.05).

^b^P = 0.02, χ^2^-test.

**Figure 1:**
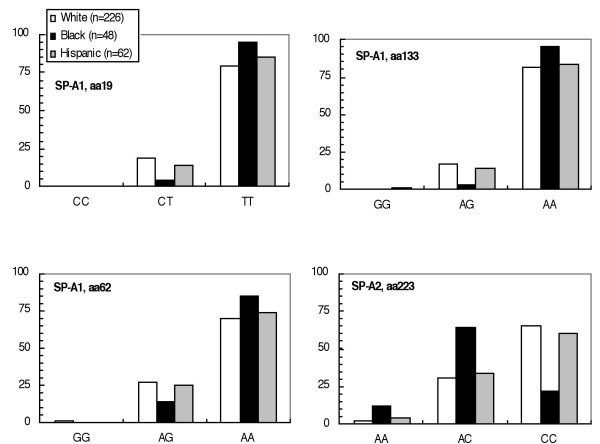
Ethnic distribution of surfactant protein A (SP-A) SNPs among infants at risk for developing asthma (P-values from χ^2 ^tests).

We did not find associations between polymorphisms within any of the SNPs and gender, maternal education, maternal allergies, maternal asthma, daycare before six months, exposure to ETS, or season of birth. Codons 19, 62, and 133 in SP-A1, and 223 in SP-A2 were associated with race/ethnicity (Figure 1). Given the association of otitis media with both race/ethnicity and specific polymorphisms within our study population, we restricted our haplotype analyses to the ethnic group with the largest number of subjects (226 white infants).

The unadjusted relationship between polymorphisms in each of the SNPs and any episode of otitis media was examined in logistic regression models in the white infants. White infants having an alanine (CT) at SP-A1 codon 19 were more than 3.8 times more likely (95% CI 1.3, 11.2) than those with a valine (TT) to have otitis media during their first year of life.

### Allele and haplotype analyses

We designated the SP-A1 and SP-A2 alleles by 6A^n ^and 1A^m ^respectively as has been previously described [21,26]. The associated nomenclature as well as the nucleotide and amino acid changes in the major SP-A1 and SP-A2 alleles are given in Table 3. The five SNPs in SP-A1 were in linkage disequilibrium. The most common SP-A1 allele haplotypes among the white infants in our study population were 6A^2^, 6A^3^, 6A^4^, and 6A in decreasing order of frequency. The four SNPs in SP-A2 were also in linkage disequilibrium (P < 0.0001). 1A^0 ^was the most common SP-A2 allele among the white infants in our study followed by 1A^1^, 1A^2^, 1A, 1A^5^, 1A^6^, and 1A^3^. All others combined made up 4% of the SP-A2 alleles.

**Table 3:** Surfactant protein A (SP-A1 and SP-A2) allele haplotypes^a ^and unadjusted odds ratios (OR) and 95% confidence intervals (CI) from logistic regression models^b ^of any episodes of otitis media before 12 months of age. (CT and MA, 1998 – 2000)

**Allele, haplotype^c^**	**Nucleotide/amino acid^d^**					**Estimated Frequency Distribution^e ^% (95% CI)**	**Any OM Episodes OR (95% CI)**
**SP-A1**	**aa 19**	**aa 50**	**aa 62**	**aa 133**	**aa 219**		
			
**6A**	C/Ala	C/Leu	G	G	C/Arg	6.1% (4.0 – 8.3)	1.96 (0.63, 6.10)
**6A**^2^	T/Val	G/Val	A	A	C/Arg	54.0% (49.3 – 58.7)	0.95 (0.44, 2.04)
**6A**^3^	T/Val	C/Leu	A	A	C/Arg	25.6% (21.6 – 29.6)	0.85 (0.45, 1.60)
**6A**^4^	T/Val	C/Leu	G	A	T/Trp	6.2% (4.1 – 8.4)	0.34 (0.15, 0.79)
**all others**						8%	
							
**SP-A2**	**aa 9**	**aa 91**	**aa 140**	**aa 223**			
			
**1A**	C/Thr	C/Pro	C	C/Gln		8.4% (6.0 – 10.8)	3.03 (0.88, 10.39)
**1A**^0^	A/Asn	G/Ala	C	C/Gln		54.2% (49.5 – 58.8)	1.04 (0.48, 2.25)
**1A**^1^	C/Thr	G/Ala	T	A/Lys		13.7% (10.7 – 16.7)	0.69 (0.34, 1.39)
**1A**^2^	C/Thr	G/Ala	C	C/Gln		10.2% (7.6 – 12.7)	0.98 (0.43, 2.22)
**1A**^3^	A/Asn	G/Ala	T	A/Lys		1.8% (0.7 – 2.9)	0.80 (0.15, 4.35)
**1A**^5^	C/Thr	C/Pro	T	C/Gln		4.6% (2.7 – 6.5)	0.38 (0.14, 1.05)
**1A**^6^	C/Thr	G/Ala	T	C/Gln		3.2% (1.7 – 4.7)	0.77 (0.20, 2.92)
**all others**						4%	

^a^N = 219 white infants with complete data for SP-A1 alleles and N = 222 white infants with complete data for SP-A2 alleles.

^b^Separate logistic regression analyses were performed for each outcome measure and each haplotype with all other haplotypes serving as the reference group in each model. All model results include estimates of model variability due to ambiguity in allele haplotype assignment (see "Data analysis" in text).

^c^By convention [ref Lofgren et al JID 2002;185:283-289], SP-A1 allele haplotypes are denoted by 6A^n ^and SP-A2 alleles by 1A^m^.

^d^Codons aa62, aa133, and aa140 are silent.

^e^Estimated frequency distribution of SP-A1 and SP-A2 haplotypes (mean, 95% CI) for white infants.

We examined associations between any otitis media during the first year of life and specific allele haplotypes in SP-A1 and SP-A2 (Table 3). The 6A^4 ^allele of SP-A1 was protective for otitis media in white infants (OR 0.34; 95% CI 0.15, 0.79). The 1A^5 ^allele in SP-A2 was underrepresented among white infants with otitis media (OR 0.38; 95% CI 0.14, 1.05). White infants with the 1A allele in SP-A2 were over 3 times more likely to have otitis media during the first year of life (OR 3.03; 95% CI 0.88, 10.39), but with wide confidence intervals that included unity.

SP-A1 and SP-A2 alleles are in close proximity and are known to be in strong linkage disequilibrium [28,29]. Within our population of white infants, the SP-A1 and SP-A2 allele haplotypes were also in linkage disequilibrium (P < 0.0001). Since the alleles at these two loci cosegregate as one unit, we examined the distribution of SP-A1 and SP-A2 alleles together. Unadjusted relationships between the SP-A haplotypes and any episode of otitis media are shown in Table 4. We did not adjust for other known otitis media risk factors (e.g. daycare, breastfeeding) because these were not associated with polymorphisms in SP-A. Consistent with our allele analysis, white infants with the 6A^4^/1A^5 ^haplotype experienced decreased risk for otitis media during their first year of life (OR 0.23; 95% CI 0.07,0.73).

**Table 4:** Estimated frequency distribution of surfactant protein A (SP-A) haplotypes among infants at risk for the development of asthma. Unadjusted odds ratios (OR) and 95% confidence intervals (CI) from logistic regression models^a ^of any episodes of otitis media before 12 months of age. (N = 226 white infants, CT and MA, 1998 – 2000.)

**SP-A Haplotype^b^**	**Estimated Frequency Distribution^c ^% (95% CI)**	**Any OM Episodes OR (95% CI)**
**6A/1A**	5.4% (3.2 – 7.2)	2.13 (0.59, 7.68)
**6A^2^/1A^0^**	49.0% (44.4 – 53.6)	1.10 (0.53, 2.30)
**6A^2^/1A^2^**	1.8% (0.6 – 2.9)	1.71 (0.20, 14.4)
**6A^2^/1A^3^**	1.0% (0.2 – 1.9)	0.90 (0.09, 8.83)
**6A^3^/1A^0^**	4.7% (2.8 – 6.6)	0.87 (0.29, 2.58)
**6A^3^/1A^1^**	11.3% (8.5 – 14.1)	0.70 (0.33, 2.47)
**6A^3^/1A^2^**	5.6% (3.6 – 7.6)	0.90 (0.33, 2.47)
**6A^3^/1A^6^**	1.7% (0.5 – 2.8)	0.73 (0.13, 4.01)
**6A^4^/1A^5^**	2.9% (1.4 – 4.4)	0.23 (0.07, 0.73)
**6A^4^/1A^6^**	1.8% (0.6 – 3.0)	0.46 (0.10, 2.09)
**Others**	14.9%	

^a^Separate logistic regression analyses were performed for each outcome measure and each haplotype with all other haplotypes serving as the reference group in each model. All model results include estimates of model variability due to ambiguity in allele haplotype assignment (see "Data analysis" in text).

^b^N = 217 infants with complete data for both SP-A1 and SP-A2 alleles.

^c^Estimated frequency distribution of SP-A haplotypes (mean, 95% CI) for white infants.

## Discussion

We found that specific SNPs, alleles, and haplotypes of SP-A were associated with otitis media risk among a cohort of infants at risk for asthma. The 6A^4 ^allele haplotype was protective for otitis media in white infants during their first year. Correspondingly, white infants with the 6A^4^/1A^5 ^haplotype experienced a 76% decreased risk for otitis media during the first year of life.

These data are in contrast to a Finnish study of the association between SP-A polymorphisms and otitis media [11]. That study reported that the 6A^4^/1A^5 ^haplotype was overrepresented among children with both acute otitis media before 6 months of age and recurrent otitis media. Under our definition of otitis media, which did not distinguish between acute otitis media and otitis media with effusion, otitis media before 6 months was highly correlated with season of birth. Among infants born in the summer or fall, one-third or more (33% and 42%, respectively) had otitis media before 6 months of age compared to less than one-quarter (23% and 18%, respectively) of the infants born in winter or spring (χ^2^, P = 0.002). Otitis media has been shown to undergo seasonal variation in parallel with respiratory virus infections [30]: rates are highest in the fall and winter months. We did not have enough power (i.e., too few subjects to stratify analysis by season of birth) to detect associations with otitis media before 6 months and specific SP-A haplotypes. In addition, by definition, our episode count for the first year of life was conservative. We identified a small number of white infants (n = 25) with 3 or more otitis media episodes during their first year, which perhaps accounts for our finding no association between recurrent otitis media and SP-A haplotypes. The population in the Finnish study was different from ours: children in the Finnish study were older (1–10 years of age), and cases were children with recurrent otitis media who were admitted to the hospital for adenoidectomy, tympanostomy, or both. The controls for the Finnish study were consecutive infants born in the hospital.

RSV infection has been strongly linked to the pathogenesis of acute otitis media [6] and polymorphisms in SP-A have been linked to susceptibility to bronchiolitis caused by this virus [31]. Lofgren et al. [31] found that 6A/1A was protective for severe RSV infection in infants (OR 0.17; 95% CI 0.04, 0.80), and the 6A^2^/1A^3 ^haplotype was associated with increased risk of disease (OR 10.4; 95% CI 1.3, 83.2). Neither of these haplotypes was associated with otitis media in our study population. Interestingly, the frequency of infants with 6A^4^/1A^5^, our protective otitis media-associated haplotype, was slightly lower among infants with RSV infections when compared to the control group (4% vs 7%) [31].

Our results indicate that SNPs at aa19 in the SP-A1 gene are associated with otitis media risk. Codon 19 is located in the N terminal domain of SP-A1. Further experiments are needed to identify whether amino acid changes in this region impact biological properties such as gene expression levels or interactions with pathogens. Infants with an alanine at codon 19 were more likely to have otitis media during their first year of life. Infants with the 6A^4^/1A^5 ^haplotype have a valine at codon19.

While the association of SP-A with otitis media is biologically plausible, otitis media is a multigenic, multifactorial disease, and there are likely other susceptibility genes involved. A genome scan mapped recurrent otitis media to chromosome q10 at marker D10S212 and to chromosome 19q at marker D19S254 [32]. SP-A is contained on chromosome 10, but is in a separate region from D10S212.

In our study, black infants experienced less otitis media than white infants. The potential problems associated with population stratification related to ethnicity have been addressed with regard to surfactant proteins [33]. Seven ethnic groups from three races (Caucasian, Black and Hispanic) were examined, and SP-A allele and genotype frequencies were shown to differ between ethnic groups from different races. We did not have enough subjects in the black (n = 47 with complete data) or Hispanic (n = 62 with complete data) groups to examine the associations of specific haplotypes with otitis media.

As stated previously, 60–80% of infants in the United States experience otitis media during the first year of life. Our finding that 73% of infants experienced otitis media during the first year is on the high end of this range. This may be due in part to the special nature of our population. All of the infants in our cohort have an older sibling in the home. Exposure to other children has been shown to be associated to otitis media [1,2]. In contrast to other studies [1-4], we did not find a strong association between otitis media and attendance in daycare outside the home. Infants in our study experienced a greater number of otitis media episodes, but the difference was not statistically significant. Another study, of 806 infants from the same cohort, identified an association between early otitis media and attendance in daycare before six months of age [24]. Therefore, the lack of statistical significance is likely due to the small number of white infants (76 total) attending daycare before six months of age.

One limitation of this study was that the cohort examined was not specifically recruited to study otitis media. We collected data by asking mothers whether their infants had a clinician-diagnosed medical condition. The diagnostic criteria may have differed between clinicians. Furthermore, mothers reported diagnosis of an ear infection but were not asked to distinguish between acute otitis media and otitis media with effusion. Different genetic polymorphisms may influence different otitis media phenotypes. Since we were unable to distinguish between different types of otitis media, grouping all types together may have diluted our findings. Since accuracy in episode counts was dependent on maternal report, we attempted to limit recall bias by asking mothers about their child's medical conditions every three months. Parents have been shown to accurately report the number of otitis media episodes (kappa = 0.94) [34]. Our study used a conservative count of otitis media episodes: two episodes occurring within the same month or consecutive months counted as one episode.

The generalizability of our results may also be limited given that our cohort contains children at risk for asthma. Since SP-A is part of the innate immune system in the lungs, infants in our cohort could differ in SP-A1 and SP-A2 hapylotype distributions compared to the general population. Liu et al. examined SP-A1 and SP-A2 allele haplotype frequencies in over 300 white Americans [33]. Their results indicate that SP-A1 allele haplotype frequencies were 56.2% 6A^2^, 24.3% 6A^3^, 9.3% 6A, 7.6% 6A^4 ^and 2.6% other. Approximate allele haplotype frequencies for SP-A2 were 53% 1A^0^, 10.2% 1A, 14.3% 1A^1^, 7.6% 1A^2^, and 14.9% all others. With the exception of allele haplotype 6A (SP-A1), the distribution of SP-A1 and SP-A2 haplotypes in our population (Table 3) is similar to that of white Americans in the general population.

Strengths of this study include the prospective study design and well characterized demographic and illness information for the infant cohort. Adjustments for uncertainty in haplotype assignment are rarely incorporated into association studies of genetic haplotypes and disease; instead these studies often use the most likely haplotype assignment [35]. We utilized logistic regression models that incorporate estimates of the effect of this uncertainty to more accurately model the association between polymorphisms in SP-A and otitis media.

## Conclusion

We identified polymorphisms within SP-A loci that are associated with otitis media in white infants at risk for asthma. These polymorphisms may play a biological role in disease, or these polymorphisms may be in linkage disequilibrium with other unmeasured markers that may be the causal polymorphism. While we cannot definitively state whether the polymorphisms are causative or are neutral markers, the results of this study suggest a role for SP-A in otitis media susceptibility. Further studies are needed to replicate, in white and other ethnic groups, our observation of a relationship between otitis media in the first year of life and polymorphisms in SP-A.

## Competing interests

The author(s) declare that they have no competing interests.

## Authors' contributions

MMP conceived of the study, analysed and interpreted data, and drafted the manuscript. JFG participated in the analysis and interpretation of data, performed the statistical analysis, and helped revise the manuscript for important intellectual content. YZ was involved in the acquisition of data and provided technical support with the genotyping. EWT helped conceive the study, and was involved in the acquisition of data. KB conceived the study, participated in its design and coordination, and helped secure funding. TRH was involved in the critical revision of manuscript for important intellectual content and provided statistical expertise. MMB was involved in the study concept and design, study supervision, critical revisions of the manuscript for intellectual content, and helped obtain funding. BPL was involved in the study concept and design, study supervision, and obtained funding. All authors read and approved the final manuscript.

## Pre-publication history

The pre-publication history for this paper can be accessed here:


